# Focal therapy will be the next step on prostate cancer management? | *Opinion: No*

**DOI:** 10.1590/S1677-5538.IBJU.2017.06.03

**Published:** 2017

**Authors:** Wagner Eduardo Matheus, Ubirajara Ferreira

**Affiliations:** 1 Departamento de Cirurgia, Departamento de Uro-oncologia, Faculdade de Ciências Médicas, Universidade Estadual de Campinas (UNICAMP), Campinas, SP, Brasil; 2 Departamento Urologia, Faculdade de Ciências Médicas da Universidade Estadual de Campinas (Unicamp), Campinas, SP, Brasil

**Keywords:** Therapeutics, Prostatic Neoplasms, Disease Management, Kidney Neoplasms

Several procedures are being described to treat prostate cancer (PCa) using minimally invasive methods (MIM), in order to achieve total cure of the disease, lower side effects and preservation of quality of life. However, we must pay maximum attention to existing scientific studies, verifying follow-up time, number of patients treated and well-designed comparative studies.

In spite of all technological advances, curative surgery is still the most important treatment for localized PCa. Alternatives to radical prostatectomy include active surveillance (for minimum volume or indolent tumors), radiotherapy and focal treatment.

Evidences obtained by randomized controlled studies show that there are very few differences among active surveillance, surgery and radiotherapy, regarding global and specific survival of low risk localized PCa in a medium follow up of 10 years. Choice of treatment by patients many times is related to urinary and rectal side effect rates presented (1–5), and cultural, economic, psychological and emotional aspects.

Focal therapies (FT) are part of the available MIM treatments for low risk PCa. Several techniques are available, including brachytherapy, cryotherapy, high intensity focal ultrasound (HIFU), interstitial laser, radiofrequency and photodynamic vascular therapy (PVT) (6). Those methods use focal energy to treat tumors obtained with low dose of radioactive substances (brachytherapy), freezing (cryotherapy), ultrasonic waves (HIFU), photothermal (intersticial laser) and action of photo-reagent drugs (PVT-Tookad) (6).

Also, FT technique may “theoretically” preserve surrounding tissues of healthy prostate, as well as neuro-vascular and sphincter structures responsible for potency and urinary continence, respectively (7). “Preliminary” results of current studies show good acceptance, low side effects and good oncologic results.

The bigger question of FT is related to the bad quality of scientific studies published: most include preliminary analysis, with low casuistic, short follow up and inadequate methodology (6).

In a systematic review (SR) recently published on FT, 43 retrospective studies were included, with low level of evidence and none randomized. In that SR, it was included 6 studies involving cryotherapy, 12 HIFU, 1 photodynamic therapy, 3 photothermal therapy, 1 radiofrequency, 1 brachytherapy guided by magnetic resonance image, and 1 with several ablation techniques, with a medium follow-up of 6 years, comprising 25 studies with 2,332 treated patients (8). Although it may seem that the number of studies and treated patients is adequate, several FT methods were used, characterizing heterogeneous groups, with short follow up period of time.

Most studies selected only patients with tumors with minimum volume, with PSA <10ng/mL, absence of Gleason 4 and 5, and low volume of disease demonstrated by histologic evaluation. This means that FT was offered to patients with very low risk tumors as an alternative to active surveillance (9).

It is fundamental to detect correctly the localization of the prostate tumor in order to perform FT. Nowadays, there is no image method totally reliable for that. Previous analysis showed that transrectal biopsy guided by ultrasound (USTR) is inaccurate to identify FT candidates and correct localization of PC. Transperineal template guided biopsy is the most recommended method to localize the disease for FT treatment, but it is an invasive method (10).

Multiparametric MR presents the needed characteristics to locate clinically significant areas of PCa. This method associated to biopsies was frequently used in FT studies, to select patients and to therapeutic planning (11, 12).

Since PCa is considered a multifocal disease in 80% of patients (13), the use of FT in only specific sites is debatable. It may not treat other significant neoplastic areas surrounding the main lesion, different from surgery and external radiotherapy, that treat the whole gland.

The concept of *index tumor* is related to the theory that only the dominant main lesion may cause progression of PCa, and distant metastasis (14). Later, this concept was modified, dividing the tumors in clinically significant lesions, with impact on longevity and quality of life, and clinically insignificant (15–17).

However, *index tumor* studies are still incipient and the theory that satellite lesions include only insignificant tumors still need more clinical evidences. Very few authors recommend treatment of *index tumor* and of clinically significant lesions.

Another great challenge for focal therapy is the definition of therapeutic success, that usually is referred as global and disease-free survival. This difficulty is observed with any treatment of PCa, since it is a disease with slow progression and with many possible subsequent treatments.

One substitute and “extrapolation” of success rates it PSA kinetics. At surgery, with complete gland removal, the ideal expected value is <0.2ng/mL. At radiotherapy, the cure and recurrence criteria are different, and PSA must be lower than 2.0ng + Nadir (lower PSA value after RT), according to P*hoenix or American Society of Radiology and Oncology (ASTRO)* criteria (18).

None of the above criteria are valid or adequate for FT, what adds confusion to disease control scenario. There is remaining prostatic tissue and the mechanism of cellular death is different in radiotherapy and immediate ablation. Therefore, PSA kinetics for FT must be different and some authors proposed the Stuttgart criteria developed for HIFU in the treatment of the whole gland: PSA value must be lower than 1.2ng/mL + Nadir following FT (19). Associated to the Stuttgart criteria, as used for brachytherapy in previous studies, it may also be considered as success the presence of velocity of elevation of PSA<0.75ng/mL per year (20).

Another controversial aspect is the evaluation of oncologic control of FT. Progression to metastatic disease is not informed in most studies, since follow-up is usually short to identify patients that develop metastasis. Cancer specific survival rates were high in published studies for the same reason. Mortality rates were also lower due to short follow up and inclusion of low risk patients (8).

One way to evaluate that aspect, also used in radiotherapy, is prostate biopsy, to verify the presence of residual disease. The results are very hard to interpret, whether with unilateral or whole gland biopsies, in clinically significant tumors or not. Positive biopsy rates were very heterogeneous due to previously cited criteria, from 0 to 17% for significant tumors, and 13-71% for all kinds of tumors (8).

Lastly, we must consider the results regarding quality of life (QL). Previous studies stressed the difficulty to perform this evaluation, since it is directly related to used question forms: quality and validation of questionnaires, data collection subjectivity and information provided by patients.

Most frequent complications of FT include urinary retention, urethral stenosis and urinary infection, rates varying from 0-17%, 0-5% and 0-17%, respectively, in five studies that reported these complications (21–25).

In the main systematic review article (SR) on FT, functional results showed a rate of 95%-100% of urinary continence, without the use of pads, and little losses in 83-100%, using only validated question forms, and very few collateral effects related to urinary continence (8).

In that same SR, erectile function evaluation was reported in 10 studies using validated question forms, showing rates of enough erectile function for penetration by 54-100% of patients, with or without the use of 5-phosphodiesterase inhibitors (5PDI). Regarding rectal toxicity, a frequent complication of RT, it was poorly described, with rates of rectal fistula varying from 0-1%, when described (8).

## CONCLUSIONS

Radical prostatectomy is still the standard treatment with better cure rates for localized PCa. Radiotherapy or brachytherapy are good alternatives for selected patients. Very low risk tumors must be submitted to active surveillance as first option. Available studies regarding FT present low level of evidences due to small number of patients, inadequate methodology, retrospective analysis and short period of follow up. Since there are still unsolved controversies, such as the existence of “index tumor”, the best evaluation of location of lesions, how to follow up such patients and how to detect failure, most FT treatments must be still be considered as experimental.

## References

[1] Wilt TJ, Brawer MK, Jones KM, Barry MJ, Aronson WJ, Fox S (2012). Radical prostatectomy versus observation for localized prostate cancer. N Engl J Med.

[2] Ficarra V, Novara G, Rosen RC, Artibani W, Carroll PR, Costello A (2012). Systematic review and meta-analysis of studies reporting urinary continence recovery after robot-assisted radical prostatectomy. Eur Urol.

[3] Ficarra V, Novara G, Ahlering TE, Costello A, Eastham JA, Graefen M (2012). Systematic review and meta-analysis of studies reporting potency rates after robot-assisted radical prostatectomy. Eur Urol.

[4] Resnick MJ, Koyama T, Fan KH, Albertsen PC, Goodman M, Hamilton AS (2013). Long-term functional outcomes after treatment for localized prostate cancer. N Engl J Med.

[5] Sheets NC, Goldin GH, Meyer AM, Wu Y, Chang Y, Stürmer T (2012). Intensity-modulated radiation therapy, proton therapy, or conformal radiation therapy and morbidity and disease control in localized prostate cancer. JAMA.

[6] Gómez-Veiga F, Martínez-Breijo S, Solsona-Narbón E, Hernández C, Ciudin A, Ribal MJ (2014). Focal therapy for prostate cancer. Alternative treatment. Actas Urol Esp.

[7] Emberton M, Gómez-Veiga F, Ahmed H, Dickinson L (2013). How will focal therapy fit in with existing treatments?. Actas Urol Esp.

[8] Valerio M, Ahmed HU, Emberton M, Lawrentschuk N, Lazzeri M, Montironi R (2014). The role of focal therapy in the management of localised prostate cancer: a systematic review. Eur Urol.

[9] Eggener SE, Scardino PT, Carroll PR, Zelefsky MJ, Sartor O, Hricak H (2007). Focal therapy for localized prostate cancer: a critical appraisal of rationale and modalities. J Urol.

[10] Hu Y, Ahmed HU, Carter T, Arumainayagam N, Lecornet E, Barzell W (2012). A biopsy simulation study to assess the accuracy of several transrectal ultrasonography (TRUS)-biopsy strategies compared with template prostate mapping biopsies in patients who have undergone radical prostatectomy. BJU Int.

[11] Nguyen PL, Chen MH, Zhang Y, Tempany CM, Cormack RA, Beard CJ (2012). Updated results of magnetic resonance imaging guided partial prostate brachytherapy for favorable risk prostate cancer: implications for focal therapy. J Urol.

[12] Napoli A, Anzidei M, De Nunzio C, Cartocci G, Panebianco V, De Dominicis C (2013). Real-time magnetic resonance-guided high-intensity focused ultrasound focal therapy for localised prostate cancer: preliminary experience. Eur Urol.

[13] Humphrey PA (1993). Complete histologic serial sectioning of a prostate gland with adenocarcinoma. Am J Surg Pathol.

[14] Bott SR, Ahmed HU, Hindley RG, Abdul-Rahman A, Freeman A, Emberton M (2010). The index lesion and focal therapy: an analysis of the pathological characteristics of prostate cancer. BJU Int.

[15] Ahmed HU (2009). The index lesion and the origin of prostate cancer. N Engl J Med.

[16] Lin D, Bayani J, Wang Y, Sadar MD, Yoshimoto M, Gout PW (2010). Development of metastatic and nonmetastatic tumor lines from a patient's prostate cancer specimen-identification of a small subpopulation with metastatic potential in the primary tumor. Prostate.

[17] Wise AM, Stamey TA, McNeal JE, Clayton JL (2002). Morphologic and clinical significance of multifocal prostate cancers in radical prostatectomy specimens. Urology.

[18] Roach M, Hanks G, Thames H, Schellhammer P, Shipley WU, Sokol GH, Sandler H (2006). Defining biochemical failure following radiotherapy with or without hormonal therapy in men with clinically localized prostate cancer: recommendations of the RTOG-ASTRO Phoenix Consensus Conference. Int J Radiat Oncol Biol Phys.

[19] Ganzer R, Robertson CN, Ward JF, Brown SC, Conti GN, Murat FJ (2011). Correlation of prostate-specific antigen nadir and biochemical failure after high-intensity focused ultrasound of localized prostate cancer based on the Stuttgart failure criteria - analysis from the @-Registry. BJU Int.

[20] Nguyen PL, Chen MH, Zhang Y, Tempany CM, Cormack RA, Beard CJ (2012). Updated results of magnetic resonance imaging guided partial prostate brachytherapy for favorable risk prostate cancer: implications for focal therapy. J Urol.

[21] Leow JJ, Heah NH, Chang SL, Chong YL, Png KS (2016). Outcomes of Robotic versus Laparoscopic Partial Nephrectomy: an Updated Meta-Analysis of 4,919 Patients. J Urol.

[22] El Fegoun AB, Barret E, Prapotnich D, Soon S, Cathelineau X, Rozet F (2011). Focal therapy with high-intensity focused ultrasound for prostate cancer in the elderly. A feasibility study with 10 years follow-up. Int Braz J Urol.

[23] Ahmed HU, Freeman A, Kirkham A, Sahu M, Scott R, Allen C (2011). Focal therapy for localized prostate cancer: a phase I/II trial. J Urol.

[24] Ahmed HU, Hindley RG, Dickinson L, Freeman A, Kirkham AP, Sahu M (2012). Focal therapy for localised unifocal and multifocal prostate cancer: a prospective development study. Lancet Oncol.

[25] Barret E, Ahallal Y, Sanchez-Salas R, Galiano M, Cosset JM, Validire P (2013). Morbidity of focal therapy in the treatment of localized prostate cancer. Eur Urol.

